# Contrasting Potato Foliage and Tuber Defense Mechanisms against the Late Blight Pathogen *Phytophthora infestans*

**DOI:** 10.1371/journal.pone.0159969

**Published:** 2016-07-21

**Authors:** Liangliang Gao, James M. Bradeen

**Affiliations:** 1 Department of Plant Pathology, University of Minnesota, St Paul, Minnesota, United States of America; 2 Department of Agronomy and Plant Genetics, University of Minnesota, St Paul, Minnesota, United States of America; 3 Stakman-Borlaug Center for Sustainable Plant Health, University of Minnesota, St. Paul, Minnesota, United States of America; National University of Singapore, SINGAPORE

## Abstract

The late blight pathogen *Phytophthora infestans* can attack both potato foliage and tubers. When inoculated with *P*. *infestans*, foliage of nontransformed ‘Russet Burbank’ (WT) develops late blight disease while that of transgenic ‘Russet Burbank’ line SP2211 (+*RB*) does not. We compared the foliar transcriptome responses of these two lines to *P*. *infestans* inoculation using an RNA-seq approach. A total of 515 million paired end RNA-seq reads were generated, representing the transcription of 29,970 genes. We also compared the differences and similarities of defense mechanisms against *P*. *infestans* in potato foliage and tubers. Differentially expressed genes, gene groups and ontology bins were identified to show similarities and differences in foliage and tuber defense mechanisms. Our results suggest that *R* gene dosage and shared biochemical pathways (such as ethylene and stress bins) contribute to *RB*-mediated incompatible potato-*P*. *infestans* interactions in both the foliage and tubers. Certain ontology bins such as cell wall and lipid metabolisms are potentially organ-specific.

## Background

*Phytophthora infestans* is a notorious plant destroyer with the capacity to attack both potato foliage and tuber. The direct costs of control efforts and lost production are estimated at over 5 billion dollars per year globally [1]. Importantly, foliage resistance against *P*. *infestans* does not guarantee tuber resistance [2], although some genetic or phenotypic correlations between tuber and foliage resistance have been reported [3, 4].

Gene *RB* (*Rpi-blb1*) [5, 6] is a disease resistance (*R*) gene conferring broad spectrum resistance against complex *P*. *infestans* races in potato foliage. Previously, we reported that higher *RB* gene copy numbers correspond to higher transcript levels and enhanced late blight resistance in the foliage [7]. Recently our research group discovered two transgenic (+*RB*) potato lines (SP2211 and SP2213) with extraordinary *RB* transcript levels that are resistant to the late blight pathogen not only in the foliage but also in the tubers in an age-dependent manner: Specifically, the *RB* gene transcript levels are highest in young tubers (post harvest) and decline as tubers age (post storage). At the same time, young tubers resist *P*. *infestans* infection but older tubers become increasingly disease susceptible. [8, 9]. Thus, the *RB*-potato-*P*. *infestans* pathosystem provides a tractable system to study how different plant organs respond to a common pathogen.

Previous transcriptome studies have documented potato foliar defense strategies against the late blight pathogen. Restrepo et al. [10] utilized a microarray technique to examine potato leaf–*P*. *infestans* interactions, highlighting a possible role for carbonic anhydrase (CA) in defining the interaction outcome. Gyetvai et al. [11] utilized the DeepSAGE method to analyze potato leaf–*P*. *infestans* interactions. That study relied mostly on assembled tags for functional analysis. Draffehn et al. [12] examined quantitative potato foliage resistance to late blight using SuperSAGE method, aligning sequence tags to the reference genome [13]. Burra et al. [14] studied the effect of phosphite treatment on transcriptome and proteome dynamics of potato and effects on disease resistance. These studies focused on how potato foliage defends against the late blight pathogen; research goals of these studies did not include comparing potato foliage and tuber responses to pathogen attack.

We published the first transcriptome analysis of potato tuber responses to *P*. *infestans* [8]. The tubers of the +*RB* transgenic line SP2211 showed increased transcription of defense related genes encoding hypersensitive induced reaction protein (HIR) and respiratory burst oxidase homolog protein B (RBOHB), and elevated transcription of defense related components such as ethylene response factors and signaling receptor kinases [8]. In the current study, we further employed RNA-seq to study transcriptome dynamics of potato foliage-*P*. *infestans* compatible and incompatible interactions. We employed whole genome sequence data from potato [13] for our analysis. We also compared potato foliage-*P*. *infestans* interactions with those of potato tuber-*P*. *infestans* interactions [8]. We identified differentially expressed (DE) genes and ontology bins that are shared components of foliage and tuber responses to *P*. *infestans* and others that are organ-specific components of potato response to pathogen attack. Our study contributes to scientific understanding of organ-specific defense responses in plants.

## Methods

### Plant materials, RNA preparation and sequencing

Nontransformed ‘Russet Burbank’ (WT) and transgenic line SP2211 (+*RB*) were examined in this study [7]. Methods for RNA-seq analysis of compatible and incompatible potato tuber-*P*. *infestans* interactions have been previously reported by Gao et al. [8]. For tuber inoculations, *P*. *infestans* sporangia were harvested from rye A plates and point inoculated on wounded whole tubers as described in Millet et al. [9].

Foliage samples were generated and collected from six week old, greenhouse-grown WT and *+RB* plants. Three WT and three +*RB* plants were each inoculated with either *P*. *infestans* US8 isolate US940480 [5] or water, providing three bio-reps for each genotype x treatment combination. *Phytophthora infestans* was maintained on Rye A medium [15] and sporangia were harvested from plates by physical scraping into distilled water. The resulting inoculum was adjusted to 1,200 sporangia/ml and incubated for 1 hour at 4 degrees Celsius and then at room temperature for 30 minutes prior to inoculation. The prepared inoculum or water (mock treatments) was sprayed onto the leaves until runoff. The greenhouse chamber was maintained at >95% humidity by frequent overhead misting. Three leaflets from each of the bio-rep plants were collected at 0 (pre-inoculation), 6, 12, 24, and 48 hours post inoculation. Collected tissue samples were immediately frozen in liquid nitrogen and stored at -80 degrees Celsius. Plants were allowed to develop disease symptoms and were visually rated on a 0–9 scale [7] 21 days after inoculation.

In total, 36 foliage samples from the two plant genotypes (WT and +*RB*) x three time points (0, 6, and 24 hpi) x two inocula (*P*. *infestans* or water) x three bio-replicates were employed for RNA extraction and RNA-seq. Total RNA was extracted from the samples using the SV Total RNA Isolation System (Promega Corporation, Madison, WI) and quantified as described earlier [8]. High quality total RNA (3ug, 50ng/μl) samples were sent to the University of Minnesota Genomics Center (UMGC) for RNA-seq library prep using the TruSeq SBS Kit (50 Cycles, paired end) and sequencing using an Illumina Hi-Seq 2000 machine.

### RNA-seq reads mapping and analysis of differentially expressed genes

RNA-seq reads were quality filtered using SolexaQA packages as described previously [8, 16]. Foliage RNA-seq raw data have been submitted to the NCBI Sequence Read Archive (accession number SRP073120). Tuber RNA-seq data were previously published and can be accessed through NCBI Sequence Read Archive accession number SRP022916 [8]. Quality filtered RNA-seq reads were analyzed using the “Tuxedo Suite” software packages as described in detail previously [8, 17].

A total of eleven pair-wise comparisons [between time (4), between lines (3), and between treatments (4)] were made using Cuffdiff of the Cufflinks software packages. Differentially expressed (DE) genes are those that showed significantly (FDR adjusted p-value <0.001) different transcript levels among comparisons, as previously described [8]. False discovery rate correction (FDR) was done using the Benjamini Hochberg method [18]. Potato gene expression fold change values were used to cluster and partition genes into groups using hierarchical clustering and the complete linkage method in Cluster 3.0 [19] and visualized in Treeview software [20] using adjusted pixel settings (threshold 1.0).

The Linux/UNIX command line options for Tophat and Cuffdiff were as previously described [8]. Custom Perl or UNIX shell scripts were used to parse the mapping/alignment results into tabular formats. The R statistical language or software environment [21] was used to generate various plots.

### MapMan ontology analysis of potato genes

We adopted MapMan (v3.5.1) ontology [22] for functional analysis of potato genes. The Mercator annotation pipeline was used to assign potato genes into functional bins by searching a variety of reference databases (http://mapman.gabipd.org/web/guest/app/mercator). A mapping file for potato is attached (S1 Table). WT and *+RB* lines were compared using the Wilcoxon rank-sum test or Fisher’s exact tests [8]. Resulting p-values from Fisher’s exact tests or Wilcoxon rank-sum tests were subjected to FDR correction using the Benjamini Hochberg method [18].

### Real-time quantitative RT-PCR for *RB* and randomly chosen genes

We used a previously established qRT-PCR protocol to determine the transcript levels of *RB* [7, 23]. *RB* gene transcript levels were normalized to *EF1α* using Qgene software [24]. RNA-seq validation, primer design (Primer Express 3.0) and RT-PCR analysis using a SYBR Green method and the ABI 7500 real time PCR machine have been described in detail previously [8].

## Results

### Gene *RB* confers resistance against late blight in the foliage; *RB* transcript levels differ between foliage and tubers

**Fig 1:**
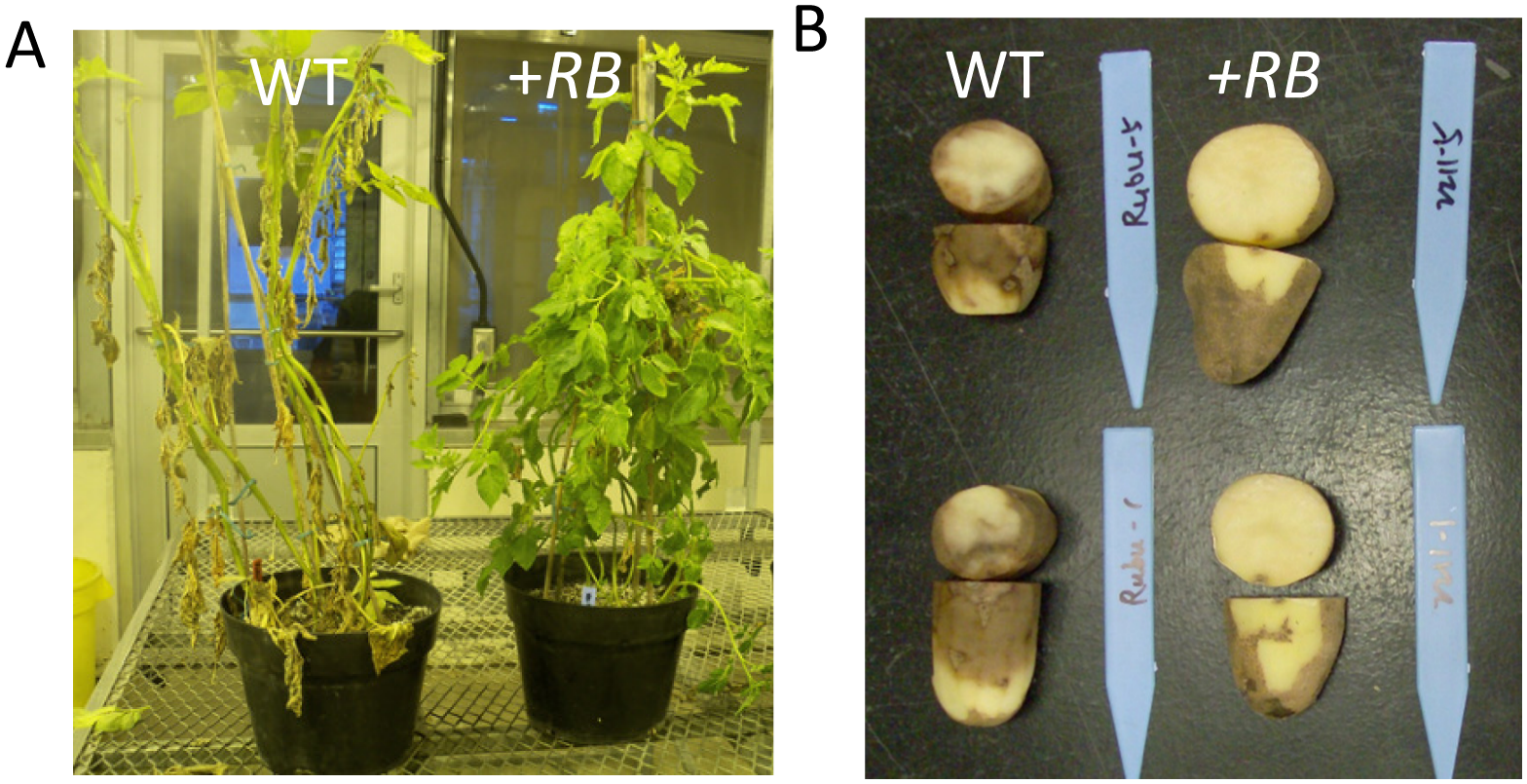
The *RB* transgene confers resistance against *P*. *infestans* in potato foliage and tubers. (A) Foliage phenotypes. Photo was taken three weeks after spray inoculation with *P*. *infestans* under moist greenhouse conditions (see methods for details) (B) Tuber phenotypes. Six week old (post-harvest) potato tuber was inoculated with *P*. *infestans*, photo was taken two weeks after inoculation (see reference [8] for details on inoculation procedures). In both cases (tuber or foliage) WT indicates nontransformed ‘Russet Burbank’, *+RB* indicates transgenic line SP2211 (‘Russet Burbank’ +*RB*), which carries 15 copies of the *RB* transgene [7].

In previous field-based evaluations, the +*RB* line examined in this study, SP2211, was ranked as the third most foliage late blight resistant of 57 transgenic lines tested and was rated as “Resistant” to foliage late blight (Fig 1) [7]. In contrast, the WT line (nontransformed ‘Russet Burbank’) was rated as “Susceptible” to foliage late blight (Fig 1) [7]. Furthermore, our research documents that the SP2211 line confers resistance to late blight not only in the foliage, but also in the tubers (Fig 1) [8, 9]. Thus, the *RB*-potato-*P*. *infestans* pathosystem provides us an opportunity to study organ-specific defense mechanisms.

**Fig 2:**
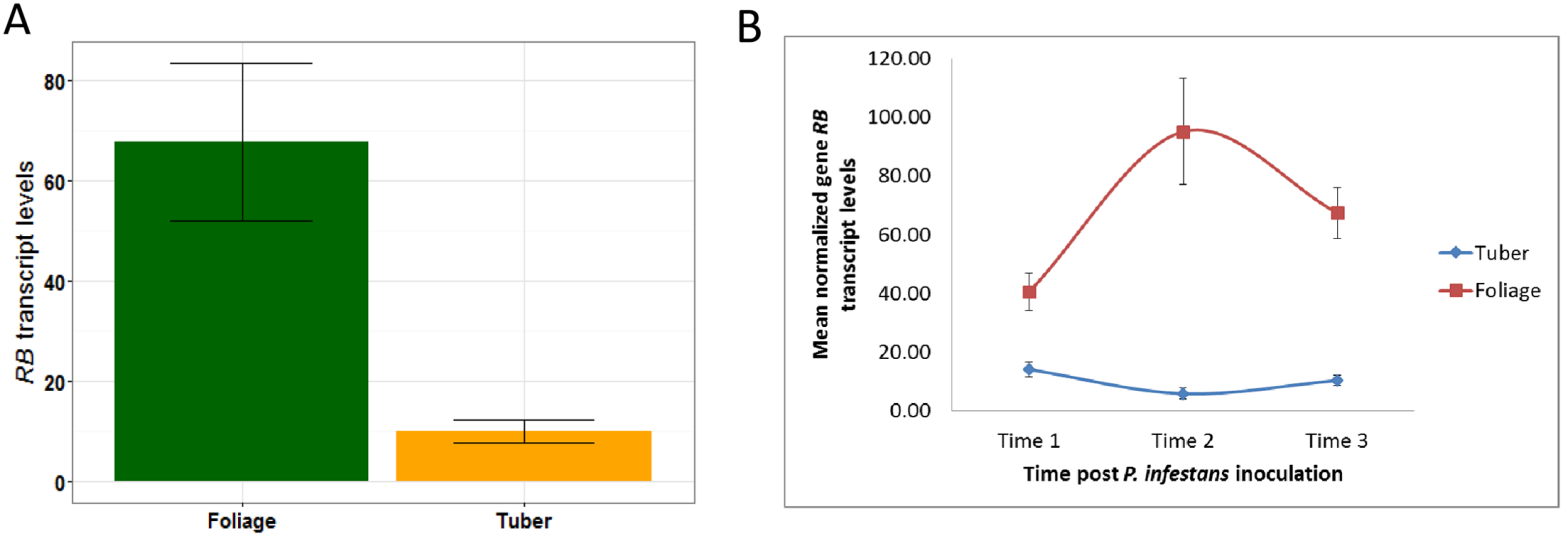
Contrasting foliage and tuber *RB* transcript levels. (A) *RB* transcript level differences between foliage and tuber averaged across select time points (0, 6 and 24 hpi for foliage; 0, 24 and 48 hpi for tubers). (B) Effects of *P*. *infestans* infection on *RB* transcript levels in foliage and tubers. Cross time point dynamics of *RB* transcript levels are presented. Time points 1, 2, and 3 indicate 0, 24, and 48 hpi for tubers and 0, 6, and 24 hpi for foliage, respectively. Y axis shows normalized *RB* transcript levels. Infection with *P*. *infestans* results in an increase in *RB* transcript levels in foliage but not in tubers.

Similar to previous research documenting that the *RB* transgene is transcriptionally up-regulated in the foliage following pathogen inoculation [25], in this study, the *RB* transgene transcript levels increased to 234% at 6 hpi and then decreased to 165% at 24 hpi in foliage. In contrast, *RB* transgene transcript levels in the tubers decreased slightly (compared to 0 hpi) in the tubers at 24 and 48 hpi (Fig 2). Interestingly, in SP2211, foliage consistently displayed higher *RB* transgene transcript levels than tubers (p<1E-4). Before pathogen inoculation, *RB* transgene transcript levels in the foliage are 2.9 fold higher than those in the tubers. After pathogen inoculation, averaged across selected time points (6 and 24 hpi for foliage; 24 and 48 hpi for tubers), *RB* transgene transcript levels in the foliage are 10 fold higher than in the tubers (81.2 for foliage vs. 8.1 for tubers). These results suggest that potato plants regulate *RB* gene transcript levels in different organs, with below ground tubers displaying less *R* gene transcript compared to above ground foliage.

### Foliar RNA-seq reads alignment to the potato reference genome sequence

From a total of 36 leaf RNA samples, 515 million paired end reads were generated, yielding an average of 14.3 million reads per sample. Around half of these reads (258.4 million or 50.1%) could be mapped uniquely to one location on the doubled monoploid (DM) potato reference genome sequence [13]. An additional 7.6 million (1.5%) reads were mapped to multiple locations within the reference genome sequence.

Across all time points studied, we detected transcription of 29,970 potato genes based on cufflinks FPKM information and gene models reported by the Potato Genome Sequencing Consortium (PGSC) [13]. The detected foliar transcripts largely overlap those detected in the tubers with 28,050 out of the 29,970 (93.6%) genes transcribed in the foliage also detected in the tubers. Importantly, 19.8% of RNA-seq reads that passed quality filters were mapped to regions outside of the current potato reference genome gene models, suggesting a need for additional genome annotation efforts. Nonetheless, qRT-PCR results correlated well with RNA-seq data with an average Pearson correlation coefficient of R = 0.87 (S3 Table). Similar to previous research findings [8, 26], our results suggest that RNA-seq and qPCR approaches can be cross-validated, confirming that the PGSC gene models (double monoploid) are appropriate for functional genomics studies on potato (tetraploid) foliage.

### Foliar and tuber transcriptomes differ dramatically; treatment (*P*. *infestans* vs. water) has a greater influence than genotype (WT vs. +*RB*) on overall transcriptome dynamics in the foliage

**Fig 3:**
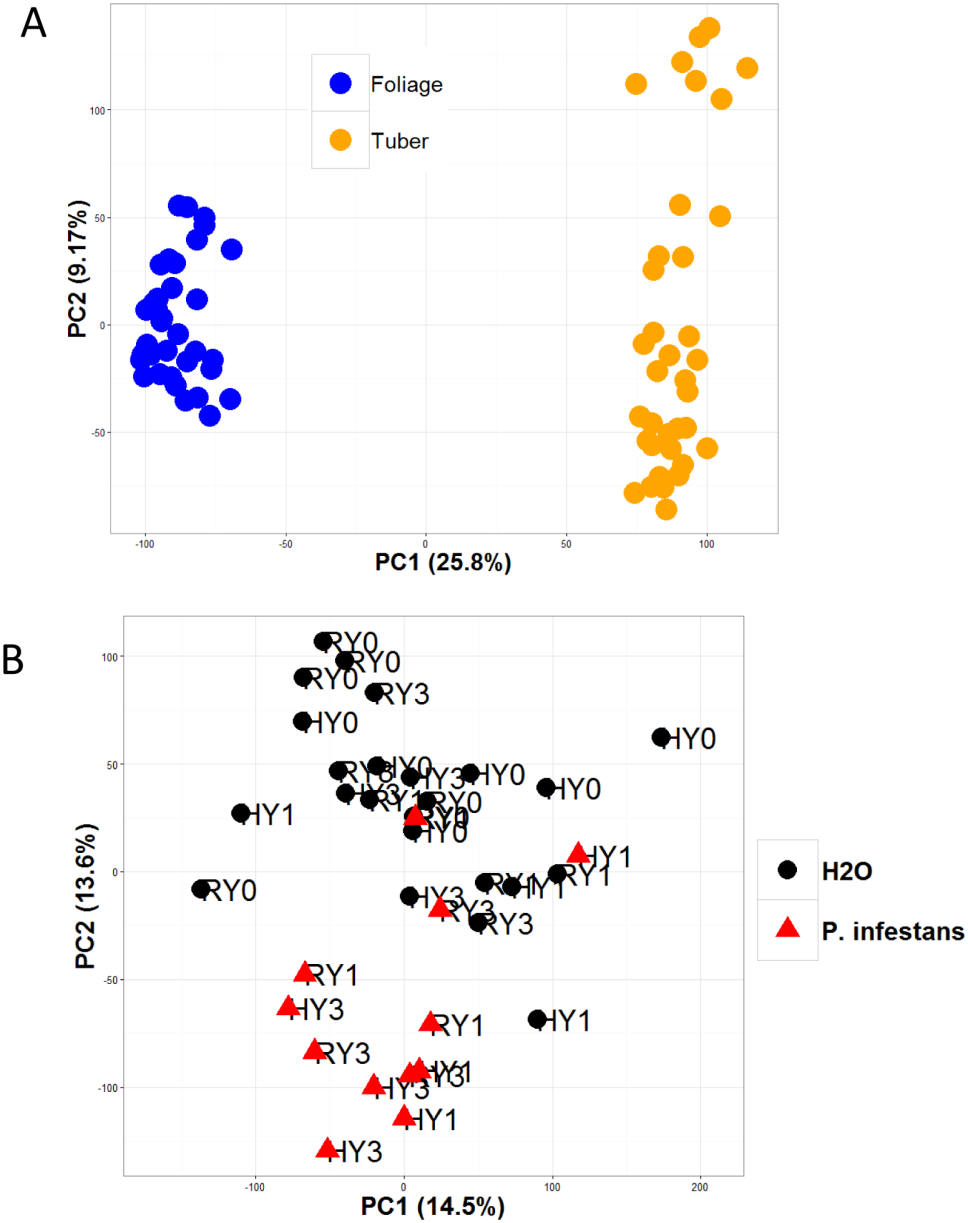
PCA analysis of foliage and tuber transcriptomes. (A) Potato foliage and tubers have distinct transcriptomes. Analysis of log2 transformed FPKM values of 31,239 genes from potato foliage and tubers. PC1 explains 25.8% of observed variation, clearly separating foliar and tuber transcriptomes. (B) WT and *+RB* foliage responds similarly to *P*. *infestans* infection. Foliage transcriptomes from water- (black circles) and *P*. *infestans-* (red triangles) inoculated samples. *P*. *infestans-*inoculated tissues were collected at 6 and 24 hpi. Sample names starting with “R” are from the nontransformed ‘Russet Burbank’ (WT) line, sample names starting with “H” are from high copy transgenic line SP2211 (+*RB*). PC2 roughly separates water- and *P*. *infestans*-inoculated samples. Neither PC1 nor PC2 separates WT from *+RB* samples.

Previously, the transcriptomes between healthy, uninfected tuber and leaf tissues were compared [27]. That same study examined *P*. *infestans-*infected foliage but not *P*. *infestans-*infected tubers. In this study, we specifically explore the transcriptomic changes induced in late blight resistant potato tubers and leaves in response to *P*. *infestans* infection. A principal component analysis (based on log2 transformed FPKM values of 31,239 genes) for foliar and tuber samples (all treatments and time points included) is shown in Fig 3A. PC1 explained >25.8% of the total variance in this dataset and clearly separates foliar and tuber transcriptomes. A principal component analysis of log2 transformed FPKM values for 29,970 genes represented in the 36 foliage RNA-seq samples is shown in Fig 3B. Together PC1 and PC2 explained >28% of the total variance of this dataset. Overall, neither time nor genotype has a clear impact on overall sample distributions in the PCA plot (separated by PC1 and PC2). Treatment (water vs. *P*. *infestans*) effects seem to be captured by PC2, with *P*. *infestans*-inoculated samples tending to be associated with negative PC2 values (less than -30, represented with red triangles in Fig 3B), and water-inoculated samples tending to be associated with positive PC2 values.

**Fig 4:**
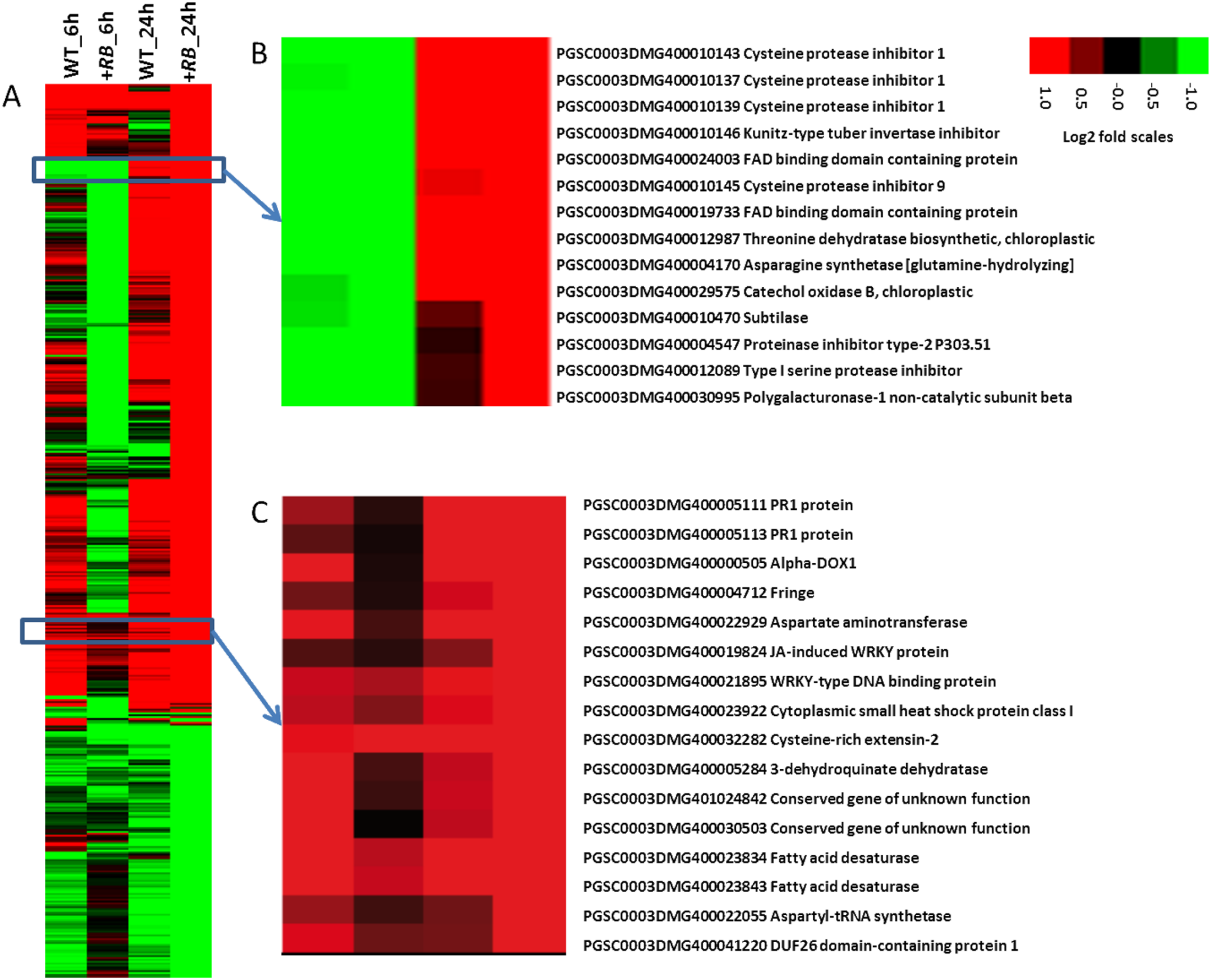
Hierarchical clustering of foliage DE genes (between treatment comparisons). Foliage of nontransformed ‘Russet Burbank’ (WT) and transgenic SP2211 (+*RB*) was inoculated with *P*. *infestans* or water. Foliage samples collected 0, 6, and 24 hpi were subjected to RNA-seq, revealing a total of 475 DE genes between water- and *P*. *infestans*-inoculated comparisons within the same genotype and the same time point. Log2(FPKM_p.inf/FPKM_mock) values were used to cluster these 475 DE genes (FDR<0.01) in Cluster 3.0 [20] using uncentered correlation and the complete linkage method. Results were visualized using Treeview [20]. Red indicates genes that are up-regulated, green indicates genes that are down-regulated. (A) Global visualization of the 475 DE genes; (B) a small gene cluster differentially regulated in +*RB* and WT at 24 hpi; (C) a small gene cluster generally up-regulated in +*RB* and WT. These clusters highlight the role of cysteine protease inhibitors and other pathogenesis related proteins (e.g., PR1) in foliar defense response to *P*. *infestans*.

In foliage, at an FDR threshold of 0.01, a total of 475 DE genes were identified in between treatment comparisons (water- vs. *P*. *infestans-*inoculated) (S4 Table). A majority (90%) of these was identified by comparison of water- and *P*. *infestans*-inoculated +*RB* samples at 24 hpi. Hierarchical clustering of the 475 DE genes revealed representative gene clusters that are known to be correlated with plant defense (e.g., *PR1* genes and cysteine protease inhibitors) (Fig 4).

**Fig 5:**
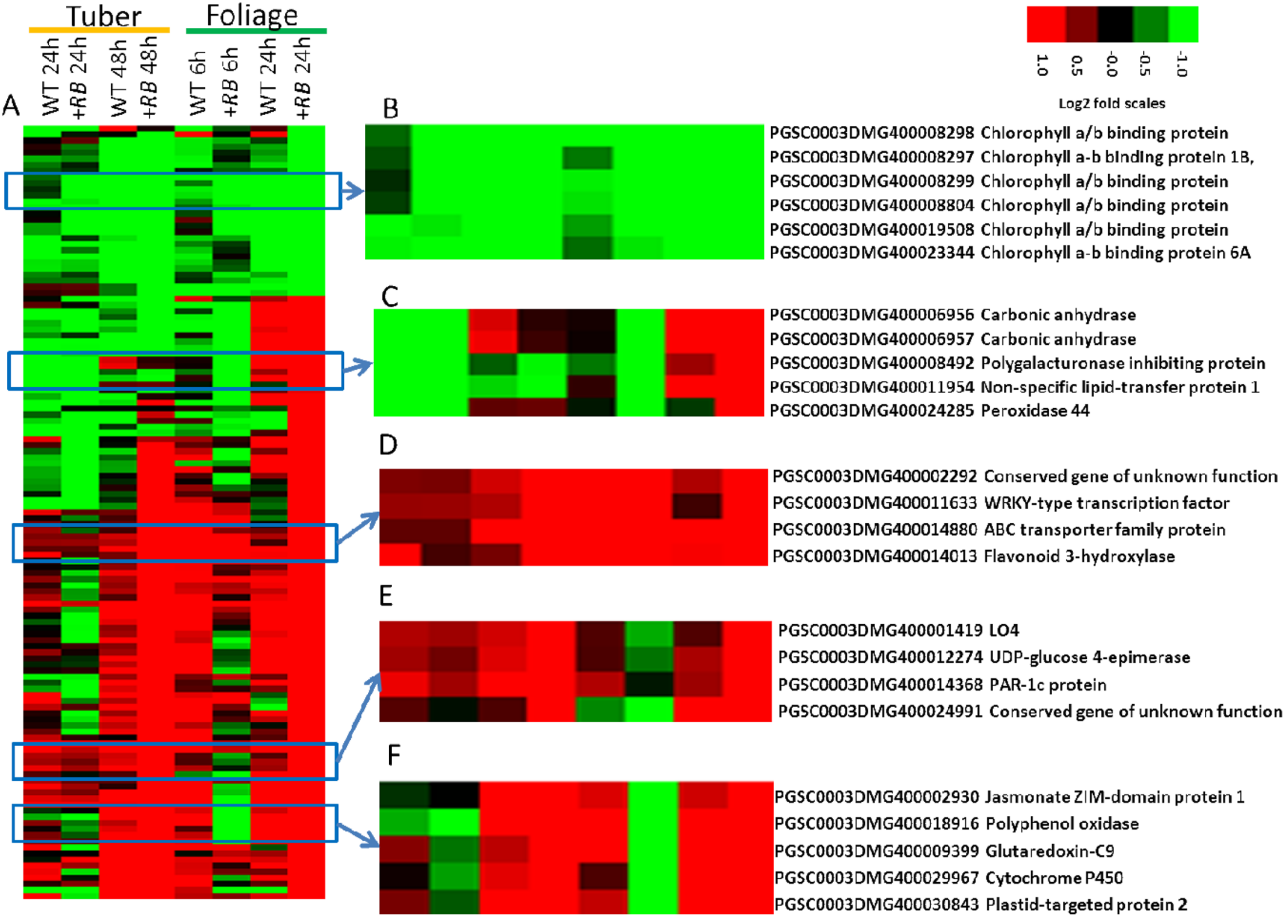
Hierarchical clustering of shared (foliage and tuber) DE genes. Foliage and tubers of nontransformed ‘Russet Burbank’ (WT) and transgenic SP2211 (*+RB*) were inoculated with *P*. *infestans* or water. Tuber samples collected at 0, 24, 48 hpi and foliage samples collected 0, 6, and 24 hpi were subjected to RNA-seq, revealing a total of 1,102 (for tubers) [8] and 475 (for foliage) DE genes between water- and *P*. *infestans-*inoculated comparisons within the same genotype and the same time point. A total of 127 DE genes are shared between both foliage (FDR<0.01) and tubers (FDR< = 0.001). Log2(FPKM_p.inf/FPKM_mock) values were used to cluster these 127 DE genes in Cluster 3.0 [20] as described in detail previously [8]. Results were visualized using Treeview [20]. Red indicates genes that are up-regulated, green indicates genes that are down-regulated. (A) Global visualization of the 127 DE genes; (B) a small cluster of photosynthesis genes down-regulated in all tissues; (C) A small gene cluster harboring carbonic anhydrase (CA); (D) a small cluster representing genes that are generally up-regulated under all conditions. (E-F) Small clusters of DE genes showing higher induction in *+RB* in both foliage (at 24 hpi) and tubers (at 48 hpi).

Among the 1,102 DE genes identified in the potato tubers [8], only 127 (S5 Table) are represented among the 475 foliage DE genes. A hierarchical clustering and heatmap visualization of the 127 shared DE genes are shown in Fig 5. The highlighted clusters indicate shared responses such as down-regulation of photosynthesis genes and up-regulation of defense related genes in both tubers and foliage.

### Foliar and tuber responses to *P*. *infestans* share some ontology bins, while other response components are organ-specific

**Fig 6:**
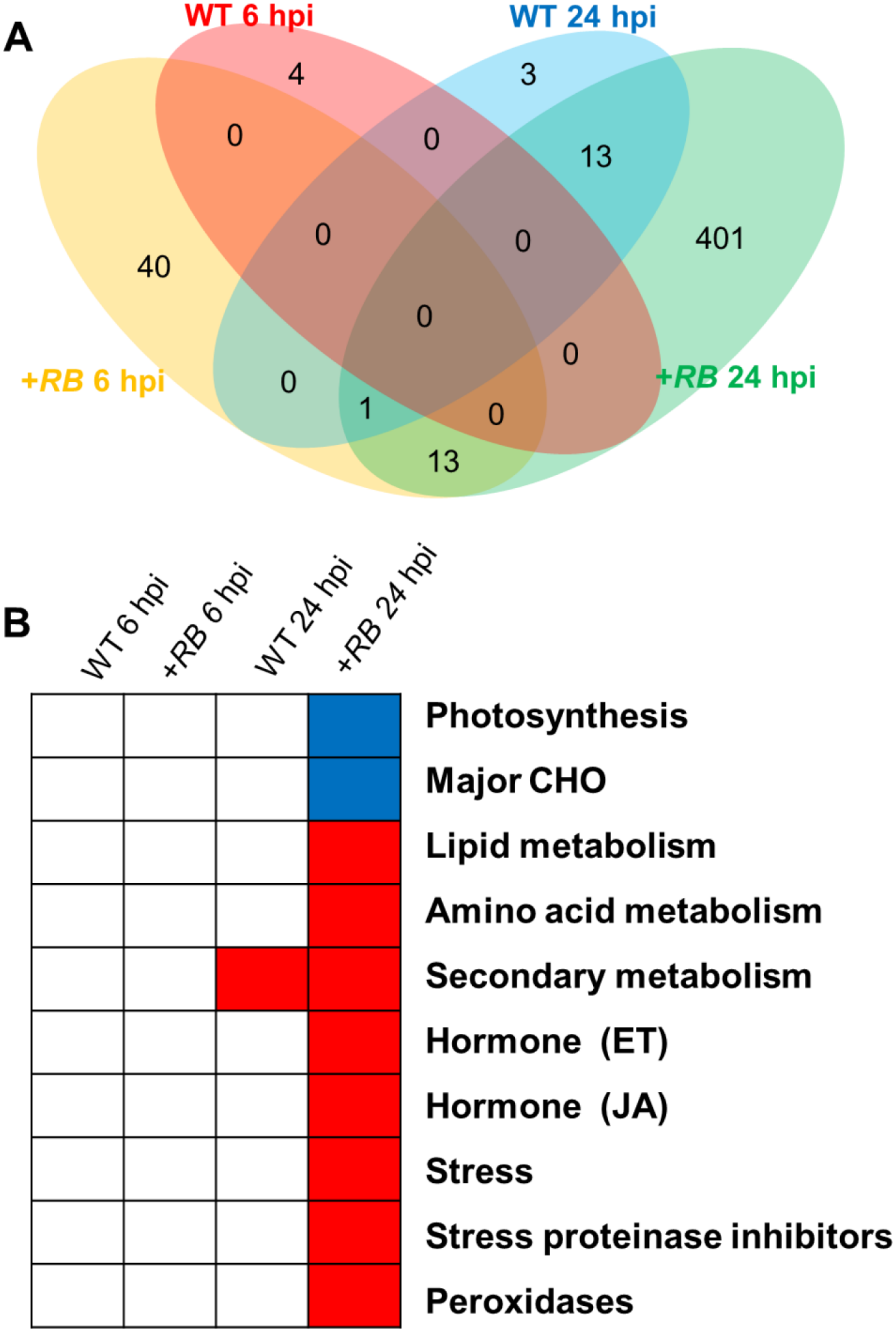
Foliage of the +*RB* line has a higher frequency of DE genes 24 hpi with *Phytophthora infestans*. Foliage samples collected 0, 6, and 24 hpi were subjected to RNA-seq, revealing a total of 475 DE genes between water- and *P*. *infestans-*inoculated comparisons within the same genotype and the same time point. (A) All 475 DE genes were analyzed using the “Venn count” function in the limma package of R [22] and results were summarized as a Venn diagram. Red: WT 6 hpi; orange: +*RB* 6 hpi; blue: WT 24 hpi; green: +*RB* 24 hpi. The results show that the *+RB* line is the main contributor of DE genes during water- vs. *P*. *infestans*-inoculated comparisons. (B) All 475 DE genes were also assigned to a MapMan ontology based on the Mercator mapping file (see methods), and subjected to Fisher’s exact test. As described previously [8], bins in red were significantly up-regulated; bins in blue were significantly down-regulated; transcription of bins in white did not change significantly. The results indicate that ontology bins encompassing ET metabolism and stress are enriched for DE genes in *+RB* but not in WT at 24 hpi.

GO enrichment analysis of the 475 foliar DE genes (derived from between treatment comparisons of water- and *P*. *infestans*-inoculated +*RB* samples at 24 hpi) reveals that ontology bins such as lipid metabolism, amino acid metabolism, secondary metabolism, ethylene (ET), jasmonic acid (JA), stress, proteinase inhibitors, and peroxidases are overrepresented by up-regulated genes in *P*. *infestans*-inoculated +*RB* samples (Fig 6). In contrast, ontology bins including photosynthesis and major CHO (carbohydrate metabolism) are overrepresented as down-regulated genes in *P*. *infestans*-inoculated +*RB* foliar samples (Fig 6). Overall, our results suggest that the +*RB* potato line shifted its metabolic prioritization from photosynthesis to pathogen defense upon *P*. *infestans* infection. Similar metabolic transition has also been documented in Arabidopsis plants under pathogen attack [28]. Interestingly, the WT samples do not show a similar transition, with few genes determined to be differentially expressed. This suggests that the WT line has failed to mount active defense components within the first 24 hours following *P*. *infestans* attack, consistent with its susceptibility to foliar late blight.

**Fig 7:**
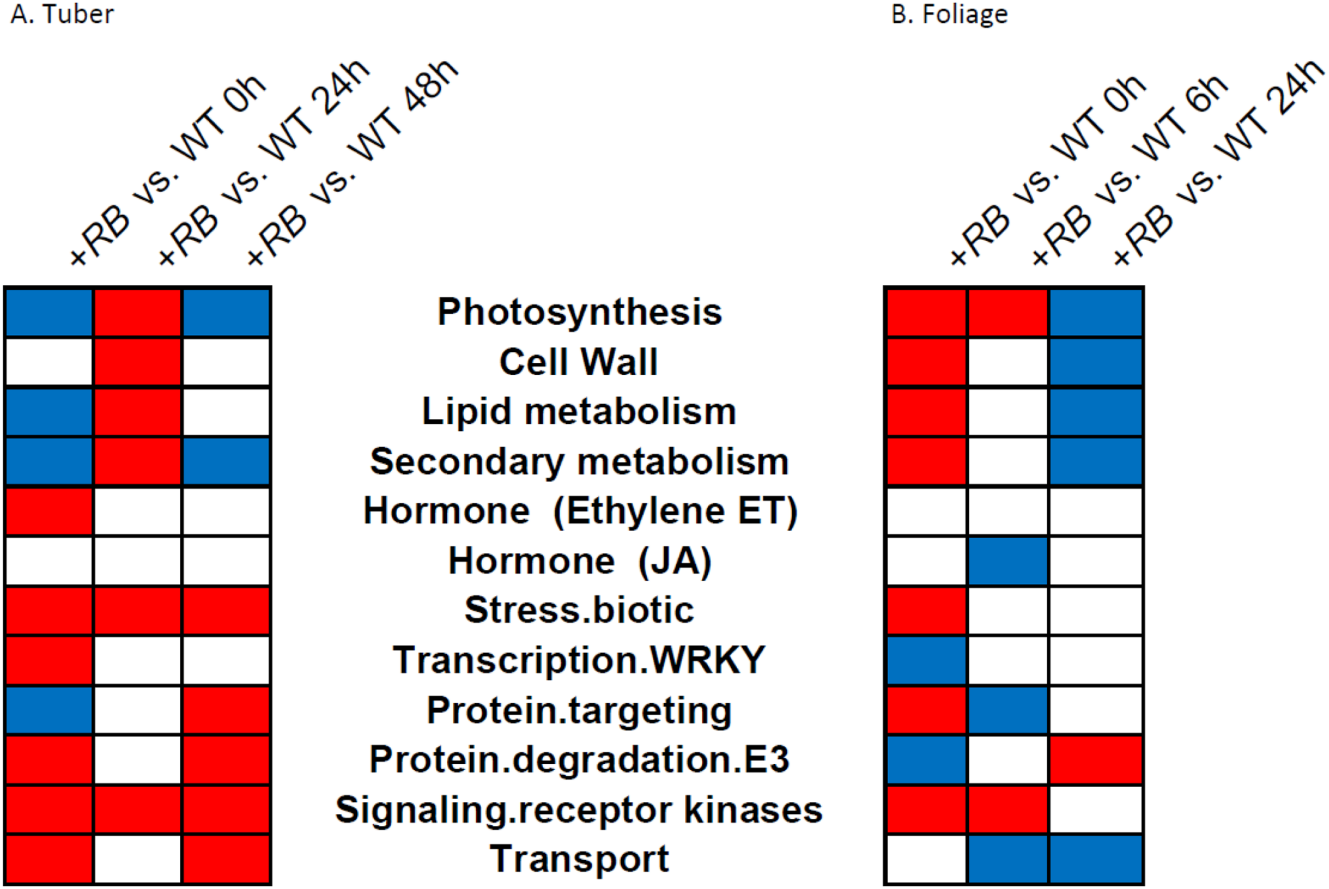
Faster and stronger activation of defense related genes or gene groups correlates with successful foliage and tubers resistance against *P*. *infestans*. Foliage and tubers of ‘Russet Burbank’ (WT) and SP2211 (*+RB*) were inoculated with *P*. *infestans* and water. We compared the RNA-seq FPKM counts for WT and +*RB* using all 39,031 gene models included in the Potato Genome Sequencing Consortium (PGSC) v3 dataset. Genes were grouped into ontology bins using a MapMan mapping file. (A) Each column represents a tuber comparison between the two genotypes at a defined time point post inoculation, as indicated. (B) Each column represents a foliar comparison between the two genotypes at a defined time point post inoculation, as indicated. As described previously [8], bins in blue are transcribed at higher levels in WT than in *+RB;* bins in red are transcribed at higher levels in *+RB* than in WT; bins in white did not significantly differ in transcript levels between WT and *+RB*. Results indicate that faster and stronger activation of defense bins, most notably biotic stress response and receptor kinase bins, occurred in tubers and foliage of the tuber late blight resistant +*RB* line.

Similar analysis in the tubers of the same genotypes in response to the same *P*. *infestans* isolate [8] revealed another set of MapMan bins that likely contribute to successful tuber defense against *P*. *infestans*. The regulation patterns of these two sets of MapMan bins (derived from foliage and tuber studies) share certain components. For example, ethylene (ET) metabolism and stress bins are highly induced both in the foliage and in the tubers of *P*. *infestans*-challenged +*RB* samples (Fig 6). At 0 hpi, when WT and +*RB* lines were compared directly using the Wilcoxon rank sum test, stress.biotic and signaling.receptor.kinases bins were transcribed at higher levels in the +*RB* line in both of the foliage and tubers (Fig 7). These ontology bins highlight potentially shared biochemical and signaling pathways in tuber and foliage defense responses.

Besides shared components, we also identified potentially organ-specific pathogen response components. For between genotype comparisons (Fig 7), lipid metabolism and secondary metabolism were transcribed at higher levels in WT (compared to +*RB*) in the tuber (at 0 hpi), but higher in the +*RB* (compared to WT) line in the foliage (at 0 hpi) (Fig 7). Cell wall components are up-regulated in the +*RB* line in the foliage but not in the tubers at 0 hpi (Fig 7). All of these differences potentially constitute organ-specific defense mechanisms against *P*. *infestans* in a potato plant.

## Discussion

### Higher *RB* gene transcript levels correlate with incompatible potato-*P*. *infestans* interactions in both the foliage and tubers

Certain previous studies documented correlation between tuber and foliar late blight resistance for particular *R* genes [4, 29]. But, in general, whether or not foliar and tuber late blight resistances are conditioned by the same gene is considered to be a function of the *R* gene itself [4, 29]. While gene *RB* has been previously described only as a foliage *R* gene [30], our previously published data revealed that *RB* can impart tuber resistance [8, 9]. In our previous study, enhanced *RB* gene transcript levels correlated with enhanced tuber disease resistance [9]. Thus transgenic (*+RB*) genotypes with low tuber *RB* transcript levels may be foliar late blight resistant but tuber blight susceptible. In contrast, transgenic lines SP2211 (examined in this study) and 2213 have comparatively high levels of *RB* transcripts even in the tuber and display late blight resistance in both foliage and tuber, in an age-dependent manner [9]. Here, we document for SP2211 that *RB* transcript levels are much higher in foliage than in tubers (Fig 2). Previously we concluded that *RB* transgene dosage correlated with both *RB* transcript levels and foliar late blight resistance [7]. Results in the current study suggest a similar correlation in potato tubers and thus we conclude that *R* gene dosage and the resulting variation in *R* gene transcript levels predict and may be determining factors in whether disease resistance is manifested in an organ-specific manner.

Pel [31] examined transcript levels for several potato late blight *R* genes (*Rpi-blb3*, *R3a*, *Rpi-vnt1*.*1*) effective in potato foliage. In almost all cases (all *Rpi-blb3* and *R3a* independently transformed lines and some *Rpi-vnt1*.*1* transformed lines), *R* gene transcript levels in the foliage were higher than in the tubers, consistent with our results (Fig 2). Furthermore, this author demonstrated that a *Rpi-blb3* line with high transgene copy numbers displayed resistance in both foliage and tubers. In the current study, line SP2211, with an estimated 15 transgene copies [7], displayed comparatively high *RB* transcript levels and resistance in both foliage and tubers. We conclude that high *R* gene transcript levels, which may be achieved by increasing copy numbers [7], is a prerequisite for conditioning tuber late blight resistance.

### Foliar and tuber transcriptomes differ dramatically but certain defense components are shared (or conserved) in both organs

Principal component analysis (Fig 3) reveals that tuber and foliar transcriptomes differ dramatically, with PC1 clearly separating the transcriptome samples into foliage and tuber clusters. A majority of DE genes is not shared between the two organs. Of the 475 DE genes (between treatment comparisons) identified in the foliage, only 127 (26.7%) are also DE in tubers.

Our ontology analysis revealed several ontology bins that are potentially organ-specific, these may include cell wall, lipid and secondary metabolisms (Fig 7). Nevertheless, we still identified ontology bins that show conserved regulation patterns across different potato organs. For example, photosynthesis is down-regulated upon pathogen infection in both foliage and tubers; stress and ethylene (ET) metabolisms were up-regulated following pathogen inoculation in both foliage and tubers (Fig 6 and [8]). The +*RB* transgenic line appears to have pre-primed defense bins, with defense response genes, including signaling receptor kinases and stress, transcribed even in the absence of the pathogen (Fig 7).

Hierarchical visualization of the regulation patterns of the 127 shared DE genes (deemed DE in both foliage and tuber) reveal groups of interesting genes (Fig 5). Both foliage and the tubers down-regulate photosynthesis related gene groups (Fig 5b), confirming the ontology bin analysis (see above paragraph). *P*. *infestans*-inoculated foliage of both WT and +*RB* displays up-regulation (compared to water-inoculated samples) of carbonic anhydrase at 24 hpi (Fig 5C). Other groups of genes (Fig 5D–5F) including WRKY transcription factors, PAR-1c protein and polyphenoel oxidases are more highly transcribed in *P*. *infestans*-inoculated (as compared to water inoculation) tuber and foliage samples (at 24 hpi for foliage, at 48 hpi for tubers).

Consistently, in both potato foliage (Fig 6) and tubers [8], faster and stronger activation of defense related ontology bins such as stress and ethylene (ET) metabolism correlates with successful defense. These results suggest that despite being different organs (below vs. above ground), the defense mechanisms in tubers and foliage could overlap. Furthermore, at a mechanistic level, the distinction between an incompatible and a compatible disease reaction, regardless of organ, results from faster and stronger activation of defense responses in the case of the incompatible reaction, with the compatible reaction demonstrating similar but slower and weaker defense responses.

## Conclusion

The current study employed molecular and transcriptomic approaches to study potato foliage-*P*. *infestans* interactions and compared the similarities and differences of potato foliage-*P*. *infestans* interactions with those of potato tuber-*P*. *infestans* interactions. DE genes, gene groups and ontology bins were identified to show potato foliage transcriptome dynamics in response to *P*. *infestans* inoculation. Shared DE genes and ontology groups between potato foliage-*P*. *infestans* and tuber-*P*. *infestans* interactions were identified (e.g. down-regulation of photosynthesis, up-regulation of ethylene (ET) and stress bins). *RB* gene transcript levels and shared ontology bins correlated with and likely directly control incompatible potato-*P*. *infestans* interactions in different plant organs.

## Supporting Information

S1 Table A MapMan mapping file for *Solanum tuberosum*. (CSV)

S2 Table qRTPCR primer sequences for randomly selected *S*. *tuberosum* genes. (CSV)

S3 Table qRTPCT and RNA-seq correlations for selected *S*. *tuberosum* genes. (CSV)

S4 Table Fold transformed FPKM values for 475 foliage DE genes. (CSV)

S5 Table Fold transformed FPKM values for 127 shared (foliage and tuber) DE genes. (CSV)
